# Endovascular treatment of intrarenal aneurysms bleeding and angiomyolipomas in a patient with tuberous sclerosis and polycystic kidney disease

**DOI:** 10.1590/2175-8239-JBN-2021-0023

**Published:** 2021-07-30

**Authors:** Túlio Leite, Lucas Vatanabe Pazinato, Maria Juliana de Aquino Vidal, Danielo de Freitas, Joaquim Mauricio da Motta Leal

**Affiliations:** 1 Universidade de São Paulo, Instituto do Coração, Departamento de Radiologia, São Paulo, SP, Brasil.; 2 Universidade de São Paulo, Faculdade de Medicina, Departamento de Radiologia, Unidade de Radiologia Intervencionista, São Paulo, SP, Brasil.; 3 Hospital Santa Genoveva, Uberlândia, MG, Brasil.; 4 Hospital Santa Genoveva, Departamento de Urologia, Uberlândia, MG, Brasil.

**Keywords:** Tuberous Sclerosis, Polycystic Kidney Diseases, Angiomyolipoma, Esclerose Tuberosa, Doenças Renais Policísticas, Angiomiolipoma

## Abstract

Tuberous sclerosis complex (TSC) and autosomal dominant polycystic kidney disease (ADPKD) are conditions related to renal failure that can rarely occur in association as a contiguous gene syndrome. Angiomyolipomas (AMLs) are renal tumors strongly related to TSC that may rupture and cause life-threatening bleedings. We present a patient with TSC, ADPKD, and renal AMLs with persistent hematuria requiring blood transfusion. The persistent hematuria was successfully treated through endovascular embolization, a minimally invasive nephron sparing technique.

## Resumo

O complexo de esclerose tuberosa (CET) e a doença renal policística autossômica dominante (DRPAD) são condições relacionadas à insuficiência renal que raramente podem ocorrer em associação como uma síndrome do gene contíguo. Angiomiolipomas (AMLs) são tumores renais fortemente relacionados ao CET que podem romper-se e causar hemorragias com risco de vida. Apresentamos um paciente com CET, DRPAD e AMLs renais com hematúria persistente que requer transfusão sanguínea. A hematúria persistente foi tratada com sucesso por meio de embolização endovascular, uma técnica de preservação do néfron minimamente invasiva.

## Introduction

Tuberous sclerosis complex (TSC) is a rare autosomal dominant neurocutaneous syndrome with involvement of multiple organs such as central nervous system, skin, renal, and lung manifestations. Although first described in the nineteenth century, its links to two suppressor genes (TSC1 and TSC2) were only identified in 1997^1^^-^ 3. The prevalence in the general population is 1:12,500, live birth rate is about 1:5,800, and about a million individuals live with this disease^3^.

Autosomal dominant polycystic kidney disease (ADPKD) is the most common genetically transmitted renal cystic disease, often presents with hypertension, abdominal pain, hematuria, and abdominal mass. ADPKD is usually bilateral and may manifest at any age but mostly appears during the fourth and fifth decades. It is also linked to mutations of two genes, PKD1 and PKD2^1^^-^ 4.

Although distinct conditions, TSC and ADPKD association is described and known as a contiguous gene syndrome involving deletion of all or part of the TSC2 and PKD1 genes. ADPKD is genetically heterogeneous, with two major genes, *PKD1* (Chr. 16.p13.3; approximately 78% families) and *PKD2* (4p21; approximately 15%), and a rare third locus, *GANAB* (11q12.3; approximately 0.3%), discovered in 2018^5^. This syndrome is characterized by clinical signs of both conditions, early-onset of renal failure in their second or third decades of life, and greater risk of malignancy^2^^,^ 4.

Angiomyolipomas (AML) are renal tumors composed of smooth muscle cells, fat, and vascular tissue in varying quantities, most being benign and asymptomatic. Although incidentally found in 0.3% of healthy adults, AMLs are strongly associated with TSC presenting an incidence of up to 80% depending on age. As they enlarge, these lesions frequently develop micro- and macro-aneurysms due to abnormal elastin-poor vascular structures, which can rupture and lead to retroperitoneal bleeding and hematuria^4^^,^ 6 ^,^ 7. Treatment of AMLs in TSC is still controversial. Nephrectomy and partial nephrectomy are invasive procedures and preserving nephrons is difficult. Laparoscopy, cryoablation, or radiofrequency are minimally invasive surgical techniques and have gained popularity^8^. More recently, selective arterial embolization has also been offered as an effective parenchymal sparing technique and preferred for treatment of AML in TSC patients. Herein, we report a case of embolization of a patient with ADPKD and TSC-related renal AMLs bleeding using an elastic polymer comprised of ethylene-vinyl alcohol copolymer (EVOH) dissolved in dimethyl sulfoxide (DMSO) with micronized tantalum powder (*Onyx* ^®^, *ev3, Irvine, CA*, USA). Informed consent was obtained from the patient for publication of the case report and accompanying images.

## Case Presentation

We report a 24-year old male patient with tuberous sclerosis and ADPKD with recurrent hematuria in treatment with tranexamic acid. This patient's TSC phenotype was severe with mental retardation and previous hydrocephalus requiring ventriculoperitoneal shunt due to TSC-related intraventricular tumors. As there was no family history, and given the autosomal dominant mode of inheritance of both TSC and ADPKD, this patient most likely represented a *de novo* case.

**Figure 1:**
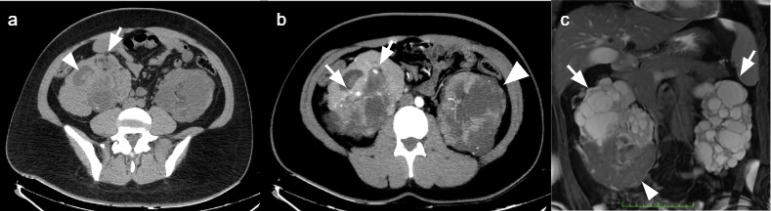
a) Non-enhanced CT evidencing multiple small masses with fat attenuation in the right kidney lower pole consistent with AMLs (arrow). Hyperattenuating material filling the dilated renal pelvis and calyces due to hematuria (arrowhead). b) Maximum intensity projection arterial phase CT evidencing pseudo-aneurysms/intrarenal aneurysms in the right kidney lower pole (arrows) and bilateral cortical cysts (arrowhead). c) Coronal T2-weighted fat-suppressed magnetic resonance image shows multiple bilateral simple cysts with thin regular walls (arrows). Renal parenchyma is shown in the lower pole of the right kidney (arrowhead).

**Figure 2:**
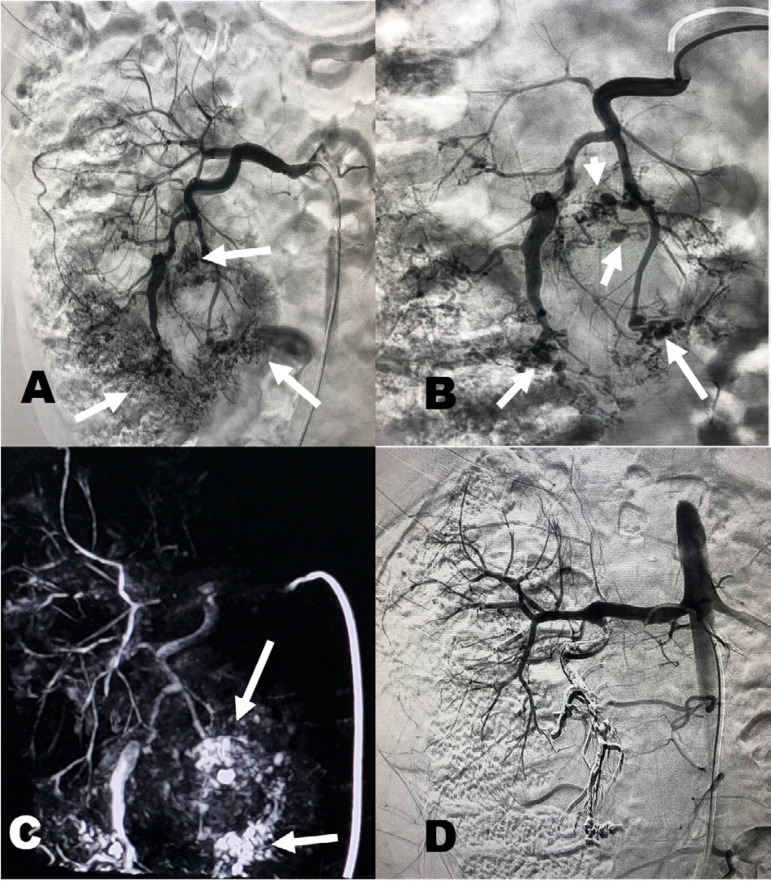
A: Arterial phase evidenced multiple saccular dilatations (aneurysms) within the inferior interlobar branches of the right renal artery (white arrow). B: Selective arteriography of the lower pole branch with multiple saccular dilations (White arrow). C: Cone beam CT was performed to assess the remaining kidney segments. In the reconstruction, a suspicious area of active bleeding was identified in the lower pole (white arrow). D: Superselective embolization of microaneurysms with Onix-18 and control arteriography with devascularization of the lower pole branches (white arrow).

He was admitted to our service as hematuria became persistent and more severe, requiring blood transfusion, but hemodynamically stable. Abdominal computed tomography showed enlarged kidneys with innumerable bilateral cysts consistent with ADPKD, small fat-containing masses in the lower pole of the right kidney consistent with AMLs and hyper-attenuating material filling the right renal pelvis due to bleeding (Figure 1). Arterial phase evidenced multiple saccular dilatations within the inferior interlobar branches of the right renal artery, suggestive of aneurysms/pseudoaneurysms (Figure 2A).

We chose to perform a right renal arteriography with a 5F Cobra catheter in which multiple microaneurysms were identified in the lower pole without signs of macrofistulas or blush (Figure 2B). Cone beam CT was performed to assess the remaining kidney segments. In the reconstruction, a suspicious area of active bleeding was identified in the lower pole (Figure 2C). We proceeded with superselective catheterization of the branches that irrigated the lower pole with a Progreat 2.4 microcatheter (Terumo) and a Fathom-16 guidewire (Boston). Superselective embolization was performed with Onix-18 (Covidien) (Figure 2D). Post-embolization angiography evidenced complete devascularization of the lesions in the lower pole while the remaining renal parenchyma was preserved. Serum creatinine before treatment was 1.9 mg/dL, rose to 2.6 mg/dL in the following day of embolization, and returned to baseline in 72 hours after hydration. Hemogram results remained stable and the patient no longer required blood transfusion. No complication occurred.

The patient was discharged after three days without hematuria and at a 1-month follow-up was asymptomatic without hematuria recurrence.

## Discussion

TSC is associated with a variable spectrum of disorders including epilepsy, intellectual disability, autistic spectrum disorder, and other neuropsychiatric problems as well as skin, heart, lung, and kidney lesions^3^. Renal complications are the second cause of death in TSC patients after neurological ones and encompasses renal cysts, angiomyolipomas, impaired kidney function and, less frequently, renal cell carcinoma^7^^,^ 9. Around 80% of TSC patients have renal angiomyolipomas that can lead to life-threatening bleeding in 25% of cases while cysts are present in approximately 30-45% of patients and may be associated with kidney failure and hypertension.^7^ In order to prevent renal complications, some authors recommend a baseline renal ultrasound before 5 years of age and a repeat every 2-3 years if results are normal, or annually if angiomyolipomas or cysts are present^9^. In summary, early molecular diagnosis of tuberous sclerosis polycystic kidney disease contiguous gene syndrome (PKDTS) may be crucial for providing appropriate disease-related surveillance and therapeutic options in patients, as well as appropriate genetic counseling for the family. ADPKD is usually inherited, but new mutations without a family history occur in approximately 10% of the cases^10^.

AMLs in TSC patients usually behave differently than in the general population as they are typically larger, bilateral, rapidly grow during childhood and adolescence, and are often associated with micro and macro-aneurysms that predispose this population to hemorrhage^8^. In fact, the most severe complication of AMLs is tumor rupture, which presents as hemodynamic shock in up to 20% of cases at the time of initial presentation. AMLs rupture is related to tumor size >4 cm, tumor growth, and aneurysm formation >5mm, the latter presenting higher specificity and sensitivity in predicting this complication^6^. Besides, lesions >4 cm are more likely to grow and to require surgical intervention^11^. The stronger relationship to aneurysm formation is represented in our case as the AMLs were small but multiple micro-aneurysms lead to hemorrhage.

Everolimus is approved for the treatment of TSC after failed tyrosine kinase inhibitor treatment. The effect of everolimus on TSC-associated AML was investigated in the EXIST-2 and extension studies, in which 6-month everolimus treatment reduced the AML volume by > 50% in 55% of patients (39 of 71) 12 ^,^ 13. In the EXIST-2 extension study, the main adverse events of everolimus were nasopharyngitis (43%), stomatitis (43%), and headache (30%). Grade 3 adverse events developed in 14% (16 of 112) of the patients^13^. Based on these results, the International Tuberous Sclerosis Complex Consensus Conference (ITSCCC) recommended the use of mammalian target of rapamycin (mTOR) inhibitors for first-line therapy for management of asymptomatic, growing angiomyolipomas >3 cm in diameter^14^.

As these patients usually have renal function impairment, nephron-sparing therapies are essential in order to delay the need for renal replacement therapy^7^. In patients with TSC and ADPKD this is even more important as they are at higher risk of renal failure. Superselective renal artery embolization is a minimally invasive alternative to renal resection that was initially reserved for symptomatic cases but has been also used on an elective basis in patients with large growing AMLs to prevent bleeding^15^. On angiography, renal AMLs frequently present hypervascular tumor with enlarged interlobar and interlobular arteries, tortuous, irregular and dilated intratumoral arteries, focal aneurysms or pseudoaneurysms, "sunburst" appearance of capillary nephrogram, "onion peel" appearance of peripheral vessels in venous phase, and no arteriovenous shunting^16^.

Numerous embolic agents have already been used including particles, coils, vascular plugs, absolute ethanol, N-butyl 2-cyanoacrylate. and Onyx^®^ (Covidien, Mansfield, MA, USA)^17^^,^ 18. Coils should be avoided because they only provide proximal vessel occlusion, which may form collaterals around or at the distal level of occlusion, making further embolization difficult or impossible. Our team's preference in these cases is a liquid agent, as it closes the distal circulation and microaneurysms avoiding recanalization and refilling by collateral circulation. Due to the variety of composition of AMLs, the response to embolization will also be different. Fat-rich AMLs have a lower response because the tissue is hypovascular and those with higher angiomyogenic component tend to respond better to embolization^16^.

The most common complication is post-embolization syndrome in up to 35.9% of patients. It occurs as a result of renal tissue necrosis and is characterized by self-limited fever, flank pain, nausea, vomiting, and leukocytosis^17^. Other complications are vascular injury, hematuria, renal infarction with abscess formation, renal failure, accidental embolization, and intraprocedural rupture^15^. In our case, no complication was encountered during embolization or post-operatively.

## Conclusion

In conclusion, AML intratumoral aneurysms are related to spontaneous rupture. Early detection and endovascular techniques to stop bleeding and reduce AML volumes are key to the preservation of renal function and long-term outcome in this population.
